# Parliament and the Profession

**Published:** 1918-04-06

**Authors:** 


### PARLIAMENT AND THE PROFESSION.

### Nerve-Strained Soldiers.

Mr. Macpherson stated that the War Office is taking steps to establish institutions for the care of nerve-strained soldiers, and medical officers are being specially trained for the treatment of this class of case. Eight are already organised, four are in process of establishment, and others are contemplated. They wlll take patients in varying numbers up to 500. The medical officers are as follows: Lieut.-Colonel Mott, 4th London General Hospital; Major Rows, Maghull Military Hospital; Major Worth, Springfield War Hospital; Lieut.-Colonel Fox, 4th Southern General Hospital, Plymouth; Major Hurst, Royal Victoria Hospital, Netley; Major Mackenzie, Glen Lomond War Hospital, Fife; Captain Culpin, 1st Southern General Hospital, Birmingham; and Captain Clements, Bradford War Hospital. The cases will be treated in military hospitals for such time as it is considered necessary to determine whether they will be fit for military service or should be discharged.

### Dispensers in Military Hospitals.

Mr. Crooks asked the Financial Secretary to the War Office whether he can now state the result of his inquiries into the position of dispensers in military hospitals as regards their claim to a war bonus, and, in reply, Mr. Macpherson stated that revised rates have been fixed as from January 1 last.

### Motor Dental Units.

Mr. Macpherson informed Mr. Pennefather that "Mobile Dental Units" are affording valuable assistance in the dental treatment of the troops in the field. The motor dental surgery presented by the Silver Thimble Fund was taken over by the War Office on March 8, and will be dispatched without delay to France.

### Nursing Inspections in Military Hospitals.

Mr. Macpherson informed Major Hill that nursing inspections are carried out by the Matron-in-Chief, Queen Alexandra's Imperial Military Nursing Service, at infirmaries and asylums used as war hospitals as well as military hospitals. Such action is taken as may be considered necessary to remedy the defects brought to notice. The Matron-in-Chief of the Joint War Committees' nurses is responsible for the inspections of the Red Cross hospitals, which are carried out systematically. Her recommendations are referred to the local County Director by the Chief County Director, who satisfies himself in each case that his directions are carried out.

### V.A.D. Efficiency Stripe.

Major Hills asked the Under-Secretary of State for War what is the effect of the red efficiency stripe recently granted to Voluntary Aid Detachment members who have completed thirteen months' service and obtained the requisite standard of efficiency. Mr. Macpherson said that such members are as far as possible given the duties of junior nurses, but must be prepared at any time to continue the ordinary duties of probationers in military hospitals. Before being eligible for the grade of assistant nurse a nursing member of a Voluntary Aid Detachment must be in possession of the red efficiency stripe, and must have completed two years' continuous service in a military, Territorial, or war hospital.

### Status of Voluntary Aid Detachments.

Major Hill inquired as to the respective responsibilities of the British Red Cross Society Headquarters, the Voluntary Aid Detachment Headquarters at Devonshire House, and the War Office with regard to the policy and organisation of the Voluntary Aid Detachment; and why the Voluntary Aid Detachment does not form an integral part of the new State Services, such as the Women's Army Auxiliary Corps, the Women's Royal Naval Service, and the Women's Air Service. Mr. Macpherson explained that the Headquarters of the British Red Cross Society are responsible for those Voluntary Aid Detachments, the organisation of which has been delegated to the Society by the Territorial Force County Association. The Voluntary Aid Detachment at Devonshire House consists of a Sub-Committee of the Joint Voluntary Aid Detachment Committee, which is responsible for the provision of Voluntary Aid Detachments required for service in military hospitals at home and abroad. Voluntary Aid Detachments on joining military hospitals, etc., for military duty come under the control of the War Office, who then assume responsibility. The scheme for the organisation of voluntary aid has been in operation for many years, and the State Services referred to are recent creations and were formed for a different purpose.

### V.A.D. Uniforms.

In reply to a question as to when it is expected that the recommendation of the Bridgeman Committee, that V.A.D. members shall receive free uniforms, will be carried out, Mr. Forster said that he understood that instructions on this subject were issued a month ago to their County Directors by the Joint Committees.

